# Hand(y) hygiene insights: Applying three theoretical models to investigate hospital patients’ and visitors’ hand hygiene behavior

**DOI:** 10.1371/journal.pone.0245543

**Published:** 2021-01-14

**Authors:** Susanne Gaube, Peter Fischer, Eva Lermer

**Affiliations:** 1 Department of Psychology, University of Regensburg, Regensburg, Germany; 2 LMU Center for Leadership and People Management, LMU Munich, Munich, Germany; 3 FOM University of Applied Sciences for Economics & Management, Munich, Germany; Jacobs University Bremen, GERMANY

## Abstract

**Background:**

Improving hand hygiene in hospitals is the most efficient method to prevent healthcare-associated infections. The hand hygiene behavior of hospital patients and visitors is not well-researched, although they pose a risk for the transmission of pathogens. Therefore, the present study had three aims: (1) Finding a suitable theoretical model to explain patients’ and visitors’ hand hygiene practice; (2) Identifying important predictors for their hand hygiene behavior; and (3) Comparing the essential determinants of hand hygiene behavior between healthcare professionals from the literature to our non-professional sample.

**Methods:**

In total *N* = 1,605 patients and visitors were surveyed on their hand hygiene practice in hospitals. The employed questionnaires were based on three theoretical models: a) the Theory of Planned Behavior (TPB); b) the Health Action Process Approach (HAPA); and c) the Theoretical Domains Framework (TDF). Structural equation modeling was used to analyze the data. To compare our results to the determinants of healthcare workers’ hand hygiene behavior, we searched for studies that used one of the three theoretical models.

**Results:**

Among patients, 52% of the variance in the hand hygiene behavior was accounted for by the TDF domains, 44% by a modified HAPA model, and 40% by the TPB factors. Among visitors, these figures were 59%, 37%, and 55%, respectively. Two clusters of variables surfaced as being essential determinants of behavior: self-regulatory processes and social influence processes. The critical determinants for healthcare professionals’ hand hygiene reported in the literature were similar to the findings from our non-professional sample.

**Conclusions:**

The TDF was identified as the most suitable model to explain patients’ and visitors’ hand hygiene practices. Patients and visitors should be included in existing behavior change intervention strategies. Newly planned interventions should focus on targeting self-regulatory and social influence processes to improve effectiveness.

## Introduction

Worldwide, healthcare-associated infections pose a severe threat to patients’ health and impose massive financial burdens on health systems [1]. The European Centre for Disease Prevention and Control estimated that 3.2 million patients were affected by healthcare-associated infections at European acute care hospitals in 2011–2012 [2]. In an earlier report, it was projected that healthcare-associated infections directly cause approximately 31.000 deaths, contribute to another 111.000 deaths, and cost about EUR 7 billion annually [3].

Improving hand hygiene behavior has been confirmed as an efficient method to prevent healthcare-associated infections. Sanitizing hands with an alcohol-based hand rub, which is the World Health Organization’s (WHO) gold standard for hospital hand hygiene in most cases, inhibits the spread of pathogens and reduces the risk of infection [4–6]. Contaminated healthcare workers’ hands are known to be the most common vehicle for the transmission of pathogens causing healthcare-associated infections [5]. However, several studies have shown that both patients and hospital visitors carry multidrug-resistant and other pathogenic organisms on their hands as well [7–9]. Therefore, non-healthcare professionals are a risk factor for the transmission of pathogens that can lead to infections. Presently, patients’ and hospital visitors’ hand hygiene behavior is not well researched. Patients and visitors are most likely an underestimated factor in infection prevention [10]. In one study, patients and their relatives were encouraged to sanitize their hands twice a day; as a result, methicillin-resistant *Staphylococcus aureus* (MRSA) infections decreased by 51% [11]. Similar positive results were found at a psychiatric facility in which patients had to sanitize their hands every four hours to prevent respiratory virus infection outbreaks [10].

Little is known about how often and in what situations patients and visitors sanitize their hands in hospitals. One reason for the deficiency of research on patients’ and visitors’ hand hygiene could be the lack of universally valid guidelines that indicate when they should sanitize their hands in healthcare facilities [12]. Four critical moments for patient hand hygiene have been proposed by one group of authors: (a) before and after touching wounds/medical devices; (b) before eating; (c) after using the restroom; and (d) when entering or leaving the patient room [13]. Another group of scholars has suggested a more comprehensive list that identifies nine moments for patient hand hygiene, including “after coughing, sneezing, or touching nose or mouth” and “before and after interacting with visitors” [12]. Visitors should sanitize their hands at least before and after contact with a patient or the patient’s surroundings [14]. Given the lack of generally accepted guidelines for patients and visitors, it is not surprising that observed hand hygiene rates vary considerably in published studies. Rates range from 0.5% of visitors at a hospital entrance hall [15] to 56.0% and 57.3% of patients and visitors, respectively, at two hospital wards [16]. One survey found that the majority of patients claimed to “always” or “usually” clean their hands after toileting (84%) and before eating (72%), but other critical moments were not reported [17]. Variations aside, all studies show that there is room for improvement. Therefore, healthcare facilities should focus on the implementation of behavior change interventions aiming to improve the hand hygiene practice of patients and visitors. Theory-based behavior change interventions are considered more effective than others [18–21], and utilizing theoretical models also provides a practicable framework for designing, implementing, evaluating an intervention. However, choosing an appropriate theory can be challenging for researchers and practitioners planning an intervention. There is an abundance of health-behavior theories, often with overlapping constructs [22, 23]. Therefore, the first goal of the present study was to find a theoretical model that effectively explains patients’ and visitors’ hand hygiene practice and that can be used to design effective behavior change interventions. We focused on three theoretical models: a) the Theory of Planned Behavior (TPB, [24]); b) the Health Action Process Approach (HAPA [25, 26]); and c) the Theoretical Domains Framework (TDF [22, 27]). These models were chosen because they have been used to study healthcare workers' hand hygiene behavior previously and are well-validated but differ substantially in complexity. Below is an introduction to the three theoretical models.

### Theoretical background

#### TPB

The Theory of Planned Behavior (TPB) is a classic health behavior theory [28] and the most widely cited model to explain hand hygiene behavior among healthcare workers [29]. For the example of hand hygiene, the TPB postulates that a person’s *intention* to clean their hands is the immediate antecedent for behavior. Moreover, the person’s intention is predicted by three variables: First, by the *attitude towards the behavior*, which is formed by beliefs about the positive and negative outcomes of performing hand hygiene, and the evaluation of these outcomes; second, by the *subjective norm*, which is shaped through perceptions about normative expectations of significant others regarding hand hygiene and a person’s motivation to comply with these expectations; and finally, by a person’s *perceived behavior control (PBC)*, which is formed through beliefs about the ease or difficulty involved in performing hand hygiene [24, 30]. A favorable attitude, salient social norm, and high degree of perceived control should lead to the intention to perform hand hygiene. However, research has shown that intention does not always translate into action, a phenomenon known as the ‘intention-behavior-gap’ [31], which is one of the main sources of criticism of the TPB [e.g., 32].

#### HAPA

To overcome the intention-behavior gap, the Health Action Process Approach (HAPA) differentiates between a *pre-intentional motivational phase*, in which intention is formed, and a *post-intentional volition phase* that leads up to action [26, 32]. In the pre-intentional phase, intention has three antecedents: *Risk perception*, which consists of the perceived likelihood of experiencing a negative outcome in relation to the behavior and the perceived severity of harm arising from the negative outcome; *outcome expectancies*, which is an assessment of the benefits and disadvantages of the action; and *perceived task self-efficacy*, which is the discerned capability of performing the behavior [26, 32]. Regarding hand hygiene, this means a person who perceives the risk of pathogen transmission as high, expects that hand cleaning reduces this risk, and believes in their capability to adhere to guidelines, would be expected to develop the intention to perform adequate hand hygiene. After the intention is formed, it needs to be reinforced by post-intentional processes. The two main processes are *planning* and, again, *self-efficacy* [32]. Two types of planning act as mediators between intention and behavior: *action planning*, which includes details about “when”, “where,” and “how” to act; and *coping planning*, which comprises strategies on how to overcome anticipated barriers to the action. Post-intentional self-efficacy can be distinguished into two beliefs: *maintenance self-efficacy*, which is comprised of optimistic beliefs about the capability to overcome barriers during the maintenance period; and *recovery self-efficacy*, which represents beliefs about the ability to regain control after a setback. This means that after the intention to perform hand hygiene is formed, the likelihood for it to translate into action increases if the person has a plan for when, where, and how they will clean their hands and how to overcome potential constraints like an empty hand-rub dispenser. The likelihood further increases if the person is optimistic about overcoming barriers and believes that compliant behavior can be restored even after a violation of the guidelines. Further *barriers (e*.*g*., *environmental constraints) and resources (e*.*g*., *social support)* can influence the intention, planning, and actual behavior. Finally, *action control*, which is comprised of self-regulatory effort, self-monitoring, and awareness of behavioral standards to adjust their behavior, is the last determinant in the volition phase [33, 34].

#### TDF

Other scholars have argued that focusing on only one theory, such as TPB or HAPA, to explain behavior is too narrow and leaves much variance unexplained [27, 35]. Relying on only one theory has two main drawbacks: First, the researcher or practitioner needs to be able to identify a theory that is relevant to the behavior out of the abundance of existing models. Second, the selected theory might miss critical theoretical domains pertinent to the action [22]. To overcome these issues, an expert team developed a consensus on which theoretical constructs are relevant for behavior change. The result is known as the Theoretical Domains Framework (TDF): a validated, integrative framework based on 33 theories and 128 constructs [22, 27]. Originally, 12 theoretical domains were identified: (1) *knowledge*; (2) *skills*; (3) *social/professional role and identity*; (4) *beliefs about capabilities (self-efficacy)*; (5) *beliefs about consequences (anticipated outcomes)*; (6) *motivation and goals (intention)*; (7) *memory*, *attention*, *and decision processes*; (8) *environmental context and resources*; (9) *social influences (norms)*; (10) *emotions*; (11) *behavioral regulations*; and (12) *nature of behavior* [22]. In a validation process, the framework was refined [27]; however, in a subsequent attempt to develop a generic TDF-based questionnaire, scholars argued for keeping the original version [36]. The framework was developed to examine the implementation of evidence-based practice, but not to ascertain “the causal processes that link theoretical constructs in a coherent explanation of behavioral regulation or behavioral change” [22: p.31]. For the hand hygiene example, this means that every domain could be relevant for predicting people’s behavior, but not all of them have to be. No formal path structure of how the domains interact to determine peoples’ behavior is proposed.

### Facilitators and barriers of hand hygiene behavior

Behavioral theories outline structural and psychological processes that can control human behavior and might be essential for changing behavior [37]. Consequently, using theoretical models to study hand hygiene behavior can show which model components are the most important facilitators and barriers toward adequate practice. An intervention to improve the behavior should then focus on those specific facilitators and barriers. Therefore, the present study's second goal was to identify critical determinants of patients’ and visitors’ hand hygiene behavior in hospitals. Previously TPB, HAPA and TDF have been used to identify facilitators and barriers of healthcare workers hand hygiene compliance. Results are reviewed briefly below.

#### TPB

A series of papers investigating relevant factors for healthcare workers’ hand hygiene behavior reported significant correlations between the three pre-intention TPB variables (attitude, norm, and PBC) and self-reported hand hygiene behavior [38–40]. In three similar studies, only subjective norm and PBC emerged as relevant predictors for self-reported hand hygiene [30, 41, 42]. At the same time, a survey among medical students found attitude and PBC, but not subjective norm, to influence self-reported compliance [43]. When objectively observing behavior instead of relying on self-reports, one study found that none of the TPB variables but only the intensity of activity in the unit was negatively associated with hand hygiene [30]. However, other scholars reported attitude and PBC to predict observed adherence [38], while yet another study found support for all TPB variables [44]. Overall, there is evidence for the relevance of all TPB model-components to predict hand hygiene behavior among healthcare workers. However, PBC emerged as being of particular importance. The construct was crucial in the studies that measured and included intention, but it never completely predicted all variance in behavior [30, 44].

#### HAPA

The PSYGIENE project implemented an intervention based on the HAPA to improve hand hygiene compliance among healthcare workers [45]. Healthcare workers completed a HAPA-based questionnaire to identify the relevant targets for the intervention. The results showed that a strong belief among staff members that hand hygiene prevents pathogen transmission was associated with high self-efficacy, high positive outcome expectations, and a strong intention to perform hand hygiene [46]. Social resources in the form of cooperation at the ward, maintenance self-efficacy, and action control were significant predictors for self-reported hand hygiene compliance among physicians. Among nurses, only action control was significantly associated with hand hygiene behavior [47]. These results indicate that post-intentional factors might play a role in overcoming the intention-behavior gap. They also show that relevant factors for engaging in a behavior can vary between target groups. Due to the project’s methodology of assessing hand hygiene behavior, it had to remain unclear how much variance in hand hygiene can be explained by the entire HAPA model.

One longitudinal study examined motivational and volitional factors for people’s handwashing, albeit outside the healthcare context [33]. They found support for the HAPA model but did not include the variables of risk perception nor barriers and resources. Self-efficacy and outcome expectancies were associated with handwashing intention. Intention, action, and coping planning were indirectly associated with handwashing via action control. However, it remains unclear if these results will translate to hand hygiene behavior in hospitals.

#### TDF

One study examined ‘real-time’ explanations for non-compliance with hand hygiene guidelines reported by healthcare staff [35] which were then coded according to the TDF. More than three-quarters of the explanations came from 3 of the 12 domains. Among these 42% belonged to the memory, attention, and decision processes domain (i.e., forgetting, being distracted or prioritizing another task), followed by 26% for the domain knowledge (i.e., lack of knowledge about guidelines), and 9% for the domain environmental context and resources (i.e., lack of time or availability of products).

Two surveys among healthcare care workers identified social/professional role and identity (i.e., what is expected of healthcare professionals), beliefs about consequences (i.e., transmission risks), and knowledge as the most important facilitators of adequate hand hygiene [48]. They also found that environmental context (i.e., time pressure, workload, and environmental controls); memory, attention, and decision processes; and beliefs about consequences to be the main barriers for compliance [48]. Another study developed and validated a TDF-based questionnaire to assess the facilitators and barriers of hand hygiene behavior among healthcare workers [49]. The authors reported that participants' self-reported hand hygiene compliance correlated with all the TDF domains measured with the instrument. So far, only one study looked at patients’ hand hygiene behavior using qualitative data from a survey and interviews, and coded the responses according to the TDF [17]. The results indicated that the four most relevant domains for patients’ hand hygiene behavior were knowledge; environmental context and resources; memory, attention, and decision processes; and social influences (i.e., social norms). It is noteworthy that forgetfulness was identified as a primary barrier for adequate hand hygiene in all three TDF-studies. However, memory and attention processes are not even included in the TPB and HAPA. This finding backs the concern that critical theoretical domains might be overlooked when relying solely on one model [22].

The third goal of the present study was to examine whether the same variables determine both healthcare professionals’ and non-professionals’ hand hygiene behavior, which is important for planning and designing interventions. Hitherto, interventions to improve hand hygiene among patients and visitors simply copied strategies used to increase hand hygiene compliance among healthcare workers. However, it cannot be taken for granted that determinants for hand hygiene are the same for both groups. Research showed that interventions tailored to its target audience are generally more effective in changing peoples’ behavior than non-tailored interventions [50–52].

### Summary of research aims

To sum up, the scope of the present paper is threefold: (1) to identify the model that can explain the most variance in hospital patients’ and visitors’ self-reported hand hygiene behavior; (2) to find critical determinants of patients’ and visitors’ self-reported hand hygiene behavior; and finally, (3) to qualitatively compare the essential determinants of hand hygiene behavior in hospitals between healthcare professionals and non-professionals. To address aims (1) and (2), we conducted surveys among hospital patients and visitors using structured questionnaires designed according to three theoretical models (TPB, HAPA, and TDF) and analyzed the data. To accomplish aim (3), we qualitatively compared our results to findings from published studies that used the same three theoretical models to study hand hygiene behavior in hospitals. The results of the present study should help hospital hygiene practitioners design and evaluate future interventions to improve patients’ and visitors’ hand hygiene behavior in hospitals and, in turn, improve patient safety.

## Methods

### Survey: Participants

Overall, the data of *N* = 1,605 patients and visitors recruited in four German hospitals were analyzed for the present study. Participants missing more than 30% of the survey items were excluded from the analysis. Thus, the data from 845 patients (age ranged 18 to 93 years; *M* = 55.72, *SD* = 16.78; 51.13% female) and 760 visitors (age ranged 18 to 91 years; *M* = 50.10, *SD* = 16.48; 56.91% female) were included in the analysis. Overall, 19 people did not report their gender, and 37 did not report their age. All participants were informed about the purpose of the study and gave informed consent. The University’s Research Ethics Committee waived the requirement for a full ethical review of the study because it was classified as low risk. The study was approved by the executive clinic management of each of the four hospitals in which the survey was conducted.

### Survey: Design and procedure

The study followed a cross-sectional design. Visitors were approached in the hospitals’ lobbies, while patients were approached in their rooms and asked to participate in the survey. The wards for the patient survey were pre-selected to ensure a diverse mix of patients. Units covered a broad range of the medical spectrum except for pediatric and palliative wards. The survey was conducted from December 2017 to November 2019. Subjects were randomly assigned to one questionnaire (TPB, HAPA, or TDF). Participants were asked to complete the survey at the hospital and hand it back to the investigators or to a nurse at the unit.

### Survey: Materials

The first section of all questionnaires was identical both for visitors and patients. It included information about the purpose of the study, instructions, and demographic questions. The questionnaires for the visitors included two items about their typical hand hygiene behavior in a hospital. The two items assessed whether they usually sanitize their hands (a) before and (b) after contact with a patient on a 5-point scale adopted from previous research [47] ranging from 1 (*always*) to 5 (*rarely*). These two moments were chosen as the dependent variable because they are suggested in the literature [14]. Moreover, one of the hospitals in which the survey was conducted placed these indications in their guidelines for visitors.

The patient questionnaire included eight items about patients’ typical hand hygiene behavior at the hospital at the following moments: (a) after entering and (b) before leaving the patient room, (c) before eating, (d) after using the restroom, (e) before and after touching wounds or medical devices, (f) before and after contact with mucous membranes, (g) after coughing or sneezing, and (h) before entering a high-risk area such as an intensive care unit. These eight indications were selected as the dependent variable because they are suggested in the literature [12]. Again, one of the hospitals in which the survey was conducted placed these indications in their guidelines for patients. The behavioral variables were assessed on a 6-point scale ranging from 1 (*always*) to 6 (*never*). The sixth option was included in the patient survey after a few participants in the precursor visitor survey mentioned there should be a *never* answer option.

All questionnaire items afterward varied depending on the theoretical model. The structure of the patient and visitor questionnaires was the same but adjusted for the target group. S1 Table includes the constructs of each questionnaire, example items, numbers of items included in each scale, scale means and standard errors, Cronbach’s alphas, and inter-item correlation for each scale and target group.

#### TPB

There is no standardized TPB-questionnaire because the theory’s author recommends constructing a new set of questions suitable for the specific behavior and population of interest. Therefore, our TPB-questionnaire was constructed according to a manual, as suggested in the literature [53]. All items were developed in English to match the manual’s recommendations, and we used forward and backward translation to maintain conceptual equivalence of the questionnaire in German. All items were pre-tested with 21 people from the general public to ensure comprehensibility and face-validity and were modified when necessary. The instrument used among visitors included 42 TPB-items and the one used among patients contained 67 TPB-items. To ensure construct validity, a Confirmatory Factor Analysis (CFA) was performed for each target group to ensure that all items included in the scales to measure the TPB model's latent variables had at least a standardized factor loading of 0.40. Items with lower standardized factor loadings were dropped, leaving 18 TPB-items among visitors and 45 among patients. The data fit well with the original four-factor TPB structure.

#### HAPA

The items for the HAPA-questionnaire were adopted from the PSYGIENE project’s HAPA-survey [54], which they had pre-tested by an independent institute. We reached out to the corresponding authors to gain access to the original German items and adjusted the questions to fit our target group and behavior. All items were pre-tested with 15 people. The visitor questionnaire contained 35 HAPA-items and the patient questionnaire contained 50 HAPA-items. Again, a CFA was performed for each target group to ensure that all items included in the scales to measure the latent variables of the HAPA models have at least a standardized factor loading of 0.40. According to the CFAs, risk perception did not load onto a single factor but should be separated into *perceived likelihood* and *perceived severity*. Among visitors, the items for the perceived likelihood variable did not load well on a factor. Therefore, we selected the most representative item to include in the model. Additionally, outcome expectancies had to be divided into positive and negative outcome expectancies, while all self-efficacy items loaded on a single factor among both groups. The resource- and inverted-barrier-items did not load on a single factor, and the barrier-items also did not load well on a separate factor. Therefore, the barriers-construct was dropped in both groups. In total, 25 items among visitors and 42 among patients were used to build the HAPA scales. The data fit sufficiently well with the suggested HAPA structure.

#### TDF

Finally, the items for the TDF-survey were adopted from a questionnaire to investigate the barriers and levers to healthcare workers’ hand hygiene behavior based on the TDF [49]. The authors developed and validated the TDF-instrument in iterative, multistep processes. Their questionnaire combined the knowledge and skills domains and dropped the nature of behavior domain. We adjusted the items to fit our target sample and behavior and used forward and backward translation to produce a German version. All items were pre-tested with 19 people from the general public. The visitor instrument consisted of 39 TDF-items, and the patient survey included 58 TDF-items. Again, CFAs for each target group were performed to ensure that all items included in the scales to measure the latent variables in the TDF models have at least a standardized factor loading of 0.40. Items with lower standardized factor loadings were dropped leaving 31 TDF-items for visitors and 46 items for patients. Among visitors, the items to measure the environmental context and resources domain did not load well on a factor; therefore, we selected the most representative item to include in the model. Overall, the data fit sufficiently well with the suggested 10-factor structure.

### Survey: Data analysis

All analyses were done in *R* version 3.5.3. The path analyses were performed using the package *lavaan* version 0.6–3 [55]. The models were fitted using a robust maximum likelihood estimation (MLF), accounting for some non-normality in the data with full information maximum likelihood (FIML) for missing data.

We defaulted to using the most commonly reported indexes and relied on cut-off levels for a good model fit suggested in the literature [e.g., 56, 57]: χ^2^ /df *≤ 2 to 3*, Root Mean Square Error of Approximation (RMSEA) < .06 to .08 with confidence intervals, Standardized Root Mean Square Residual (SRMR) *≤* .08, Comparative Fit Index (CFI) ≥ .95, and Tucker Lewis Index (TLI) ≥ .95. Also, we used the Akaike Information Criterion (AIC) and Bayesian Information Criterion (BIC) for model comparison, where the rule ‘the smaller, the better’ applies. If the majority of these fit indexes imply a good fit, we consider the model to fit the data well. To address the first research aim, we compared the model fit indices and the amount of variance in self-reported hand hygiene behavior explained by each theoretical model among both hospital patients and visitors. To attain the second research objective, we tested which variables of the three theoretical models correlate statistically significantly with self-reported hand hygiene behavior or intention among both target groups. The study’s pre-registration, data, and R-script will be made available online upon publication: https://osf.io/m2v56/

### Qualitative comparison: Search strategy and eligibility criteria

To address the third and final research aim, we conducted a literature search on Google Scholar, PsycINFO, and Web of Science from the year 2000 up to March 2020 for relevant articles in the English language. Additionally, the reference lists of eligible publications were screened. We considered any article that measured the variables of at least one of the three theoretical models (TPB, HAPA, and TDF) to study healthcare workers’ observed or self-reported hand hygiene behavior. Both quantitative and qualitative studies were included in the review as long as they were published and peer-reviewed. We did not assess the quality of the studies nor their risk of bias. The search strategy included the following keywords: hand hygiene, hand hygiene compliance, hand washing, healthcare workers, healthcare professionals, physicians, nurses, theory of planned behavior, TPB, health action process approach, HAPA, theoretical domains framework, TDF, theory, behavioral theory.

## Results

### Findings self-reported hand hygiene behavior

**Fig 1:**
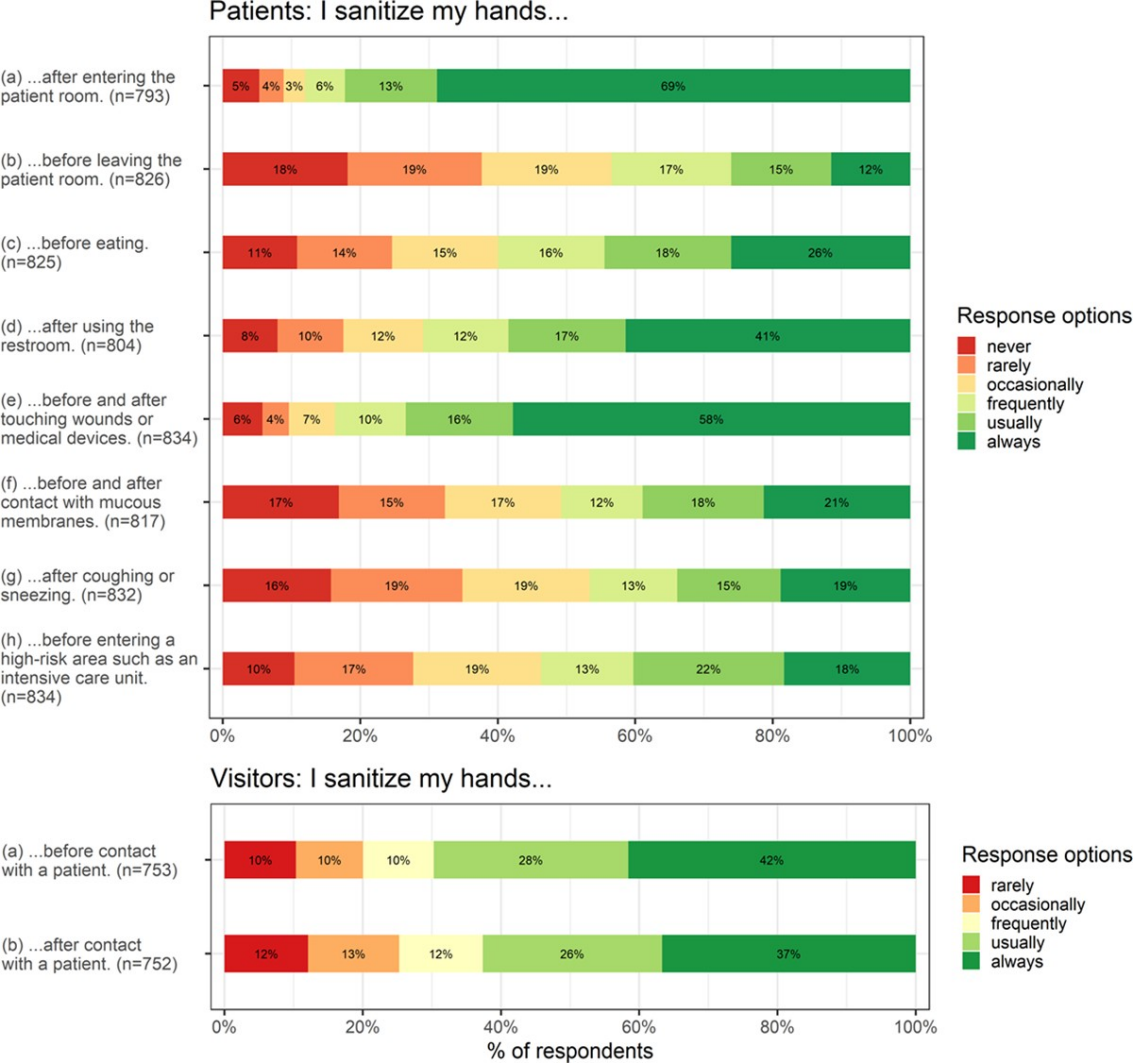
Frequency distribution of self-reported hand hygiene behavior in hospitals.

The patients’ mean level of self-reported hand hygiene behavior was *M* = 4.08 (*SD* = 1.24), and the visitors’ mean level of self-reported hand hygiene behavior was *M* = 3.71 (*SD* = 1.28). Overall, both patients and visitors reported *frequently* sanitizing their hands in the hospital when averaging their respective indications for hand hygiene (eight indications for patients and two for visitors). Fig 1 shows the participants’ responses separated for the indications surveyed.

### Findings aim one: Identifying a suitable behavioral model

#### TPB

The hypothesized TPB path structure fitted well with the data from both patients (χ^2^ = 5.25, df = 2, χ^2^/df = 2.63, *p* = .072, RMSEA = .08 with 90%-CI [.00, .16], SRMR = .02, CFI = .99, and TLI = .97) and visitors (χ^2^ = 4.02, df = 2, χ^2^/df = 2.01, *p* = .134, RMSEA = .06 with 90%-CI [.00, .16], SRMR = .02, CFI = .99, and TLI = .98). Attitude, subjective norm, and PBC accounted for 52% of the variance in patients’ and 53% of the variance in visitors’ behavioral intention. Intention and PBC explained 40% of the variance in self-reported hand hygiene behavior among hospital patients and 55% among hospital visitors.

#### HAPA

The initially hypothesized HAPA path structure was neither a good fit for the patient data (χ^2^ = 83.14, df = 11, χ^2^/df = 7.56, *p* < .001, RMSEA = .16 with 90%-CI [.13, .19], SRMR = .05, CFI = .80, TLI = .57, AIC = 2375.12, and BIC = 2442.40) nor the visitor data (χ^2^ = 173.66, df = 11, χ^2^/df = 15.79, *p* = < .001, RMSEA = .24 with 90%-CI [.21, .27], SRMR = .08, CFI = .47, TLI = -.16, AIC = 2735.68, and BIC = 2803.11). Consequently, the models were modified post hoc. Thinking about the nature of hand hygiene behavior, hospital patients most likely do not engage in considerable planning for “when”, “where”, and “how” to clean their hands, nor for how to overcome any anticipated barriers to performing hand hygiene. Therefore, the planning construct was removed from the models. Additionally, the action control variable was allowed to correlate both with the self-reported hand hygiene behavior as well as intention, since the correlation between these variables was high and the modification improved the model fit substantially. This is also supported by previous research [33]. The new models fitted well with both the patient data (χ^2^ = 8.63, df = 4, χ^2^/df = 2.16, *p* = .071, RMSEA = .07 with 90%-CI [.00, .13], SRMR = .02, CFI = .99, TLI = .95, AIC = 1332.98, and BIC = 1386.10) and the visitor data (χ^2^ = 3.06, df = 4, χ^2^/df = 0.77, *p* = .547, RMSEA = < .001 with 90%-CI [.00, .08], SRMR = .01, CFI = 1.00, TLI = 1.00, AIC = 1559.36, and BIC = 1612.60). The new models outperformed the original models significantly (both *p* < .001). Self-efficacy, positive and negative outcome expectancies, risk perception (likelihood and severity), environmental resources, and action control jointly accounted for 52% of the variance in patients’ and 49% of the variance in visitors’ behavioral intention. Self-efficacy, intention, environmental resources, and action control together explained 44% of the variance in self-reported hand hygiene behavior among hospital patients and 37% among hospital visitors.

#### TDF

The hypothesized TDF models were just-identified (i.e., equal numbers of variables and parameters with a unique solution). Therefore, the models fitted the data perfectly, and theoretically, there is no need to report fit indices. Nevertheless, we fixed a non-significant parameter (environmental context and resources) to zero to compare the model fit of the three theoretical models. The modified TDF path structure fitted the data very well among both patients (χ^2^ = 1.70, df = 1, χ^2^/df = 1.70, *p* = .192 RMSEA = .05 with 90%-CI [.00, .18], SRMR = .01, CFI = 1.00, and TLI = 0.96) and visitors (χ^2^ = 1.57, df = 1, χ^2^/df = 1.57, *p* = .211, RMSEA = .05 with 90%-CI [.00, .19], SRMR = .01, CFI = 1.00, and TLI = 0.97). The parameter estimates for these two models can be found in the S2 Table. All social-cognitive variables together explained 52% of the variance in self-reported hand hygiene behavior among patients and 59% among hospital visitors.

### Findings aim two: Detecting critical determinants

#### TPB

**Fig 2:**
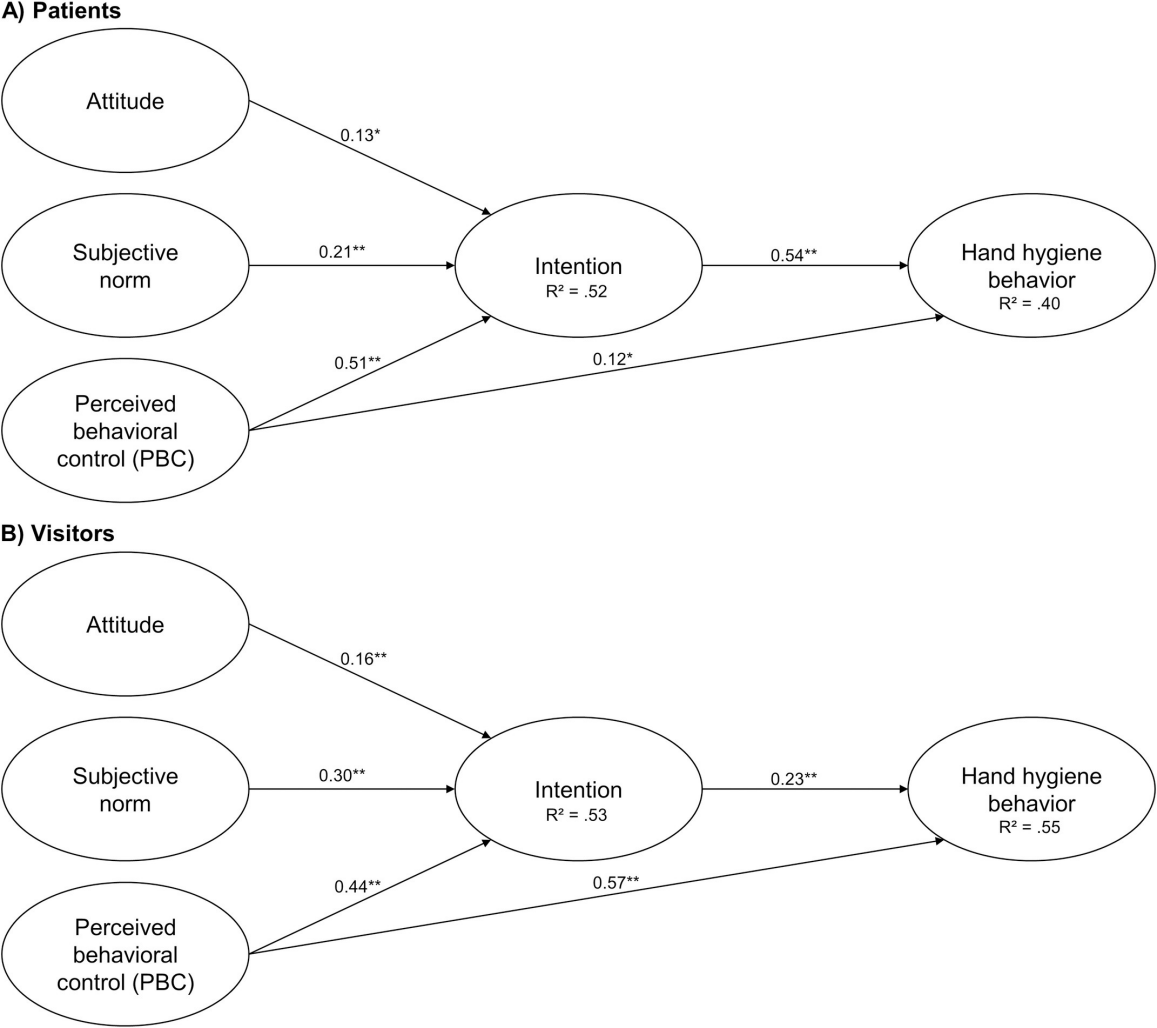
TPB path models with standardized parameter estimates to predict hand hygiene behavior. Note: * *p* < .05, ** *p* < .001, *n*_*(total patients)*_ = *n*_*(used patients)*_ = 286, *n*_*(total visitors)*_ = 251, *n*_*(used visitors)*_ = 248.

In both samples, all three pre-intentional TPB-variables (attitude, subjective norm, and PBC) significantly correlated with people’s intention to sanitize their hands, which in turn correlated significantly with self-reported behavior. Fig 2 shows the parameter estimates with corresponding standard errors and confidence intervals displayed in Table 1.

**Table 1:** Coefficients for the TPB path models.

						95% CI for β	
	Path	β	*SE*	*z*	*p*	LL	UL
Patients	Attitude → Intention	0.13	0.04	3.04	.002	0.05	0.21
	Subjective Norm → Intention	0.21	0.05	4.46	< .001	0.12	0.30
	PBC → Intention	0.51	0.04	13.05	< .001	0.44	0.59
	Intention → Behavior	0.54	0.05	10.03	< .001	0.44	0.65
	PBC → Behavior	0.12	0.06	2.06	.040	0.01	0.23
Visitors	Attitude → Intention	0.16	0.04	3.77	< .001	0.07	0.24
	Subjective Norm → Intention	0.30	0.05	5.63	< .001	0.20	0.41
	PBC → Intention	0.44	0.05	8.35	< .001	0.33	0.54
	Intention → Behavior	0.23	0.04	5.64	< .001	0.15	0.32
	PBC → Behavior	0.57	0.05	12.35	< .001	0.48	0.66

*Note*. β = standardized coefficient, *SE* = standard error; *z* = β /*SE*, *p* = probability value, LL = lower limit, UP = upper limit.

#### HAPA

**Fig 3:**
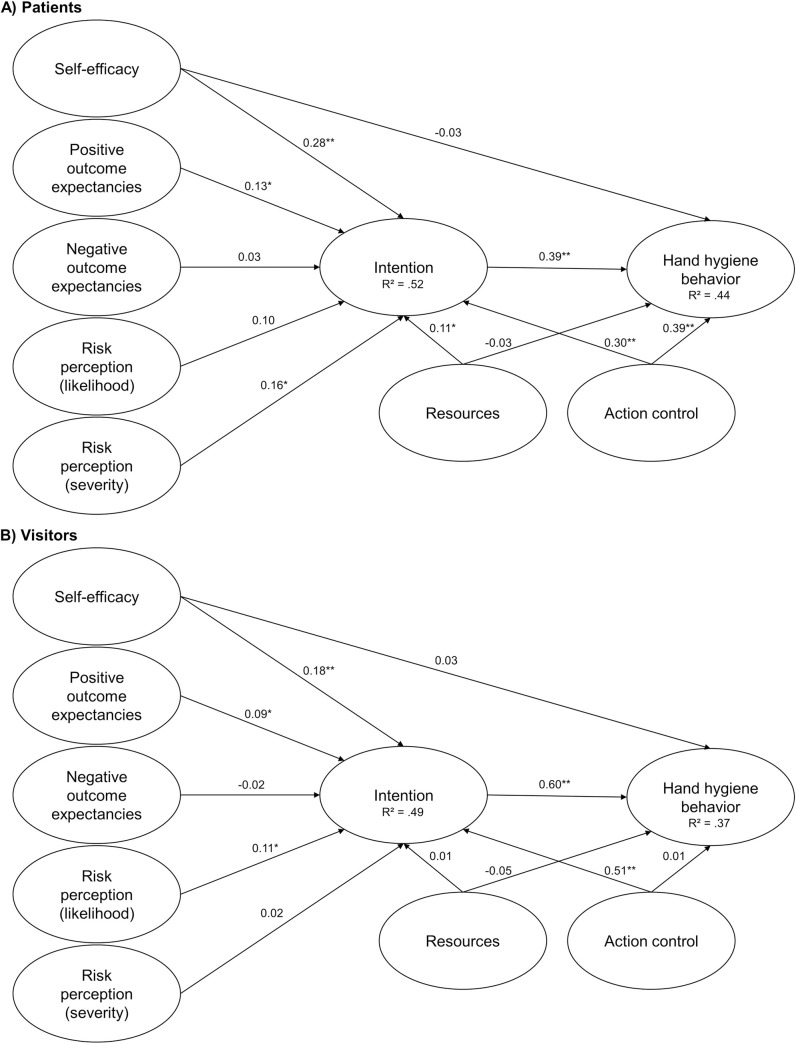
HAPA path models with standardized parameter estimates to predict hand hygiene behavior. Note: * *p* < .05, ** *p* < .001, *n*_*(total patients)*_ = 266, *n*_*(used patients)*_ = 255, *n*_*(total visitors)*_ = 264, *n*_*(used visitors)*_ = 257.

For patients, self-efficacy, positive outcome expectations, perceived severity of harm, environmental resources, and action control were all significantly correlated with intention. Negative outcome expectations and perceived likelihood of experiencing a negative outcome did not considerably influence intention. Hand hygiene behavior correlated positively with intention and action control. Among visitors, self-control, positive outcome expectations, and action control were significantly correlated with the intention to clean their hands before and after patient contact. The perceived likelihood of an adverse outcome but not the perceived severity was associated with intention. Negative outcome expectations and environmental resources did not influence intention. The only significant correlates for hand hygiene behavior among visitors was intention. Fig 3 shows the standardized parameter estimates for the adjusted HAPA model with corresponding standard errors and confidence intervals displayed in Table 2.

**Table 2:** Coefficients for the HAPA path models.

						95% CI for β	
	Path	β	*SE*	*z*	*p*	LL	UL
Patients	Self-Efficacy → Intention	0.28	0.04	7.02	< .001	0.20	0.36
	Pos. Out. Exp. → Intention	0.13	0.06	2.17	.030	0.01	0.25
	Neg. Out. Exp. → Intention	0.03	0.06	0.54	.587	-0.09	0.16
	Likelihood → Intention	0.10	0.06	1.70	.089	-0.01	0.21
	Severity → Intention	0.16	0.05	3.47	.001	0.07	0.25
	Resources → Intention	0.11	0.04	2.37	.018	0.02	0.19
	Action Control → Intention	0.30	0.04	6.77	< .001	0.21	0.38
	Self-Efficacy → Behavior	-0.03	0.05	-0.52	.602	-0.13	0.07
	Intention → Behavior	0.39	0.06	6.36	< .001	0.27	0.51
	Resources → Behavior	-0.03	0.06	-0.48	.632	-0.14	0.08
	Action Control → Behavior	0.39	0.06	6.95	< .001	0.28	0.50
Visitors	Self-Efficacy → Intention	0.18	0.05	3.48	< .001	0.08	0.28
	Pos. Out. Exp. → Intention	0.09	0.04	2.32	.020	0.01	0.17
	Neg. Out. Exp. → Intention	-0.02	0.07	-0.29	.769	-0.15	0.11
	Likelihood → Intention	0.11	0.05	2.37	.018	0.02	0.20
	Severity → Intention	0.02	0.05	0.37	.713	-0.09	0.12
	Resources → Intention	0.01	0.05	0.17	.868	-0.10	0.11
	Action Control → Intention	0.51	0.04	12.50	< .001	0.43	0.59
	Self-Efficacy → Behavior	0.03	0.05	0.54	.592	-0.07	0.12
	Intention → Behavior	0.60	0.07	9.11	< .001	0.47	0.73
	Resources → Behavior	-0.05	0.06	-0.78	.436	-0.16	0.07
	Action Control → Behavior	0.01	0.07	0.11	.911	-0.13	0.15

*Note*. β = standardized coefficient, SE = standard error; z = β /SE, p = probability value, LL = lower limit, UP = upper limit.

#### TDF

**Fig 4:**
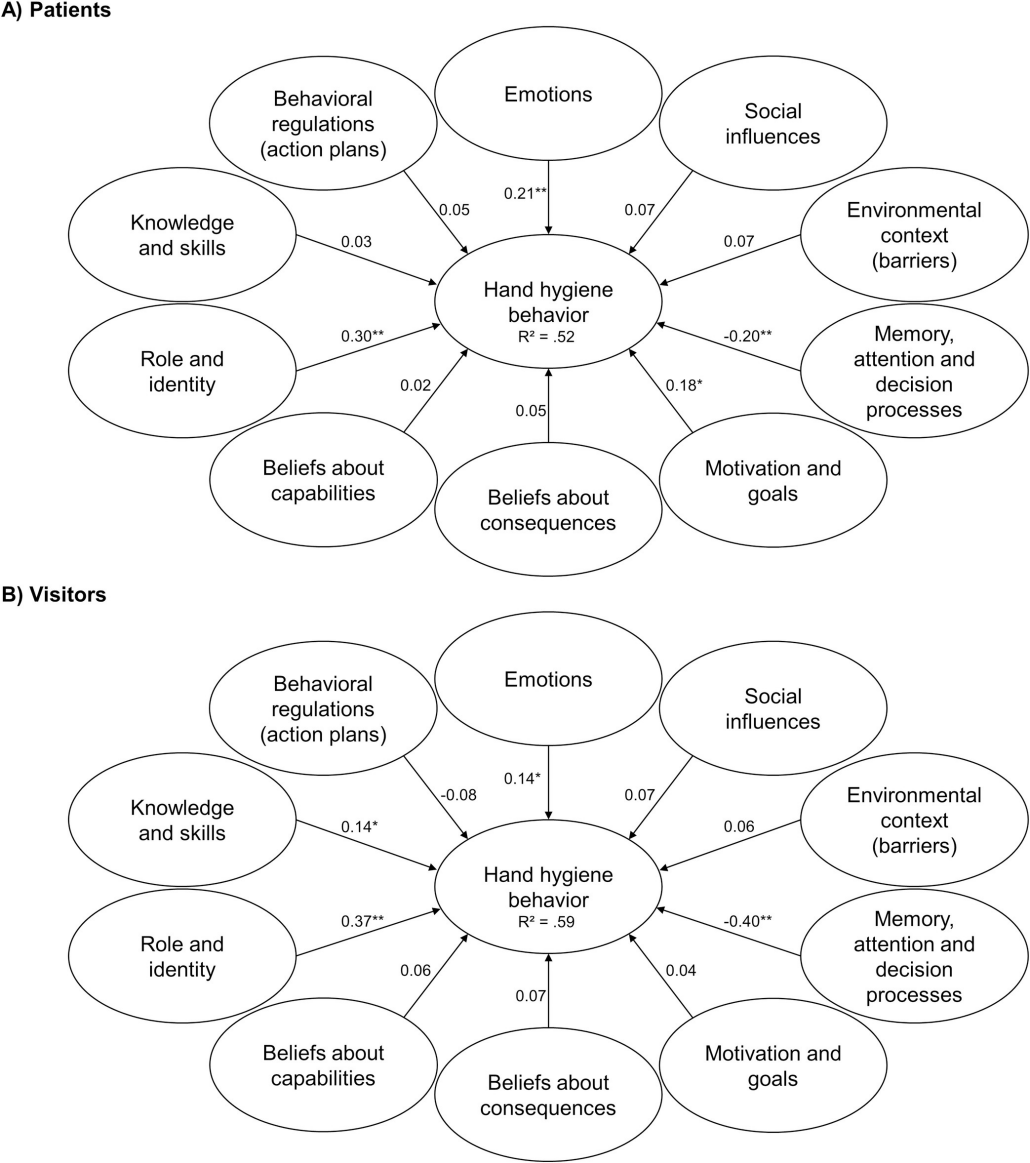
TDF path models with standardized parameter estimates to predict hand hygiene behavior. Note: * *p* < .05, ** *p* < .001, *n*_*(total patients)*_ = 293, *n*_*(used patients)*_ = 273, *n*_*(total visitors)*_ = 245, *n*_*(used visitors)*_ = 238.

For patients, role and identity, motivation and goals, memory, attention, and decision processes as well as emotions significantly correlated with self-reported hand hygiene behavior. For visitors, the significant predictors were role and identity, memory, attention, and decision processes, knowledge and skills, as well as emotions. Consequently, the only difference was that instead of motivation and goals, knowledge and skills were associated with behavior, but both variables were only weak predictors. Fig 4 shows the standardized parameter estimates for the TDF model with corresponding standard errors and confidence intervals displayed in Table 3.

**Table 3:** Coefficients for the TDF path models.

						95% CI for β	
	Path	β	*SE*	*z*	*p*	LL	UL
Patients	Knowledge/Skills → Behavior	0.03	0.05	0.61	.540	-0.07	0.13
	Role and Identity → Behavior	0.30	0.04	6.81	< .001	0.21	0.38
	Capability → Behavior	0.02	0.05	0.45	.656	-0.08	0.12
	Consequences → Behavior	0.05	0.06	0.81	.415	-0.06	0.15
	Motivation/Goals → Behavior	0.18	0.06	3.05	.002	0.06	0.30
	Memory/Attention → Behavior	-0.20	0.05	-3.82	< .001	-0.31	-0.10
	Environment → Behavior	0.07	0.05	1.33	.184	-0.03	0.16
	Social Influences → Behavior	0.07	0.05	1.34	.179	-0.03	0.17
	Emotions → Behavior	0.21	0.06	3.56	< .001	0.10	0.33
	Beh. Regulations → Behavior	0.05	0.06	0.83	.406	-0.07	0.17
Visitors	Knowledge/Skills → Behavior	0.14	0.05	2.74	.006	0.04	0.23
	Role and Identity → Behavior	0.37	0.06	5.89	< .001	0.25	0.49
	Capability → Behavior	0.06	0.06	1.08	.279	-0.05	0.17
	Consequences → Behavior	0.07	0.05	1.41	.160	-0.03	0.18
	Motivation/Goals → Behavior	0.04	0.05	0.83	.409	-0.06	0.15
	Memory/Attention → Behavior	-0.40	0.05	-7.79	< .001	-0.50	-0.30
	Environment → Behavior	0.06	0.05	1.26	.209	-0.03	0.15
	Social Influences → Behavior	0.07	0.05	1.56	.119	-0.02	0.17
	Emotions → Behavior	0.14	0.06	2.54	.011	0.03	0.25
	Beh. Regulations → Behavior	-0.08	0.05	-1.63	.103	-0.18	0.02

*Note*. β = standardized coefficient, *SE* = standard error; *z* = β */SE*, *p* = probability value, LL = lower limit, UP = upper limit.

### Findings aim three: Comparing patients and visitors with healthcare professionals

The literature search yielded twelve studies that met the eligibility criteria (see Table 4). Eight studies used the TPB, only one employed the HAPA, and three studies deployed the TDF to identify determinants for hand hygiene behavior among healthcare workers. Most studies included in the qualitative comparison reported self-reported hand hygiene behavior as the outcome measure. In contrast, only two studies used observed hand hygiene compliance, and another two studies described both self-reported and observed behavior. Two studies included only nursing staff, and another two studies encompassed only medical staff, while the sample of all other studies was comprised of both groups of healthcare workers.

**Table 4:** Summary of studies used for the comparison.

Theory	Study	Year	Participants	Outcome variables
TPB	O'Boyle et al. [30]	2001	Nursing staff	Self-reported and observed hand hygiene
	Pittet et al. [44]	2004	Medical staff and medical students	Observed hand hygiene
	Pessoa-Silva et al. [41]	2005	Nursing and medical staff	Self-reported hand hygiene
	Whitby et al. [40]	2006	Nursing staff	Self-reported hand hygiene
	Sax et al. [42]	2007	Nursing and medical staff	Self-reported hand hygiene
	McLaws et al. [39]	2012	Nursing staff, nursing students, and medical students/trainees	Self-reported hand hygiene
	Eiamsitrakoon et al. [38]	2013	Nursing and medical staff	Self-reported and observed hand hygiene
	Erasmus et al. [43]	2020	Medical students	Self-reported hand hygiene
HAPA	von Lengerke et al. [47]	2015	Nursing and medical staff	Self-reported hand hygiene
TDF	Dyson et al. [49]	2013	Nursing, medical, and other healthcare staff	Self-reported hand hygiene
	Fuller et al. [35]	2014	Nursing, medical, and other healthcare staff	Observed hand hygiene
	Smith et al. [48]	2019	Nursing and other healthcare staff	Self-reported hand hygiene

*Note*. Other healthcare staff includes professions like physiotherapists, personal support workers, and ancillary staff, among others.

#### TPB

Our results corresponded with several of the published studies in which the three pre-intentional variables attitude, subjective norm, and PBC were shown to be relevant predictors for self-reported hand hygiene compliance among healthcare workers [38–40]. Other studies found only two out of three pre-intentional variables to be important for self-reported behavior [30, 41–43]. All studies that used the TPB to predict self-reported hand hygiene behavior identified PBC as being critical. Studies that used observed hand hygiene behavior as an outcome variable also found significant correlations with PBC [38, 44]. Only two studies measured intention and showed that the construct was crucial, but it did not predict all variance in behavior, which is known as the ‘intention-behavior-gap’. We saw a similar effect in our analysis. An overview of the comparison can be found in Table 5.

**Table 5:** Support for the association between individual determinants and hand hygiene behavior.

		Target group		
Theory	Variable	Patients^1^	Visitors^1^	Healthcare workers^2^
TPB	Attitude	*	**	5/8 [38–40, 43, 44]
	Subjective norm	**	**	7/8 [30, 38–42, 44]
	PBC	**	**	8/8 [30, 38–44]
	Intention	**	**	2/2 [30, 44]
HAPA	Self-efficacy	**	**	1/1 [47]
	Outcome expectancies	*	*	*Not tested*
	Risk perception	*	*	*Not tested*
	Intention	**	**	*Not tested*
	Resources and barriers	*	ns	1/1 [47]
	Action and coping planning	ns	ns	0/1
	Action control	**	**	1/1 [47]
TDF	Knowledge and skills	ns	*	3/3 [35, 48, 49]
	Social/professional role and identity	**	**	2/3 [48, 49]
	Beliefs about capability	ns	ns	1/3 [49]
	Beliefs about consequences	ns	ns	2/3 [48, 49]
	Motivation and goals	*	ns	1/3 [49]
	Memory, attention, decision processes	**	**	3/3 [35, 48, 49]
	Environmental context and resources	ns	ns	3/3 [35, 48, 49]
	Social influences (norms)	ns	ns	1/3 [49]
	Emotions	**	*	1/3 [49]
	Behavioral regulations	ns	ns	1/3 [49]

*Note*. ^*1*^Results from the present study; ns = not significant

* *p* < .05

** *p* < .001 with a link either directly to behavior or intention; ^2^Results from previously published work; number of studies that found support for a variable out of total number reviewed (e.g., 5/8 five out of eight studies), with citation of the studies that found support in superscript.

#### HAPA

To our knowledge, only one study connected HAPA variables with hand hygiene behavior among healthcare workers [47]. This study found that self-reported hand hygiene compliance among physicians was associated with environmental resources, maintenance self-efficacy, and action control. Self-reported behavior among nurses was only linked to action control. The paper did not report the pre-intention HAPA variables (risk-perception, outcome expectancies, and task self-efficacy). When only considering the post-intention variables, the patients’ and visitors’ results correspond to the findings among healthcare workers. Action control correlated significantly with patients’ hand hygiene behavior, and the link between action control and visitor’s hand hygiene behavior was mediated through intention.

#### TDF

Three studies examined the determinants of hand hygiene behavior among healthcare workers using the TDF [35, 48, 49]. All three found memory, attention, and decision processes (i.e., forgetting, lack of focus, or prioritizing other tasks) to be among the most crucial barriers to adequate hand hygiene, which is in accordance with our results on the behavior of patients and visitors. A second important determinant for healthcare workers’ hand hygiene compliance in all three studies was knowledge. In our analysis of patients and visitors, this factor only correlated with visitors’ hand hygiene behavior significantly, and the effect was not very profound. A third domain deemed imperative for healthcare workers’ hand hygiene compliance in the published studies was environmental context and resources, which did not emerge in our analysis of patients and visitors as a significant predictor. Two of the three studies identified social/professional role and identity as a central determinant for healthcare workers’ hand hygiene behavior, which is corresponds with our results. Finally, we did not find evidence that beliefs about consequences were a good predictor for hand hygiene behavior among patients and visitors, while two of the three studies that investigated healthcare workers’ hand hygiene compliance found that this variable was a relevant predictor of behavior for the studied population.

## Discussion

### Summary aim one: Identifying a suitable behavioral model

The first goal of the present study was to identify a theoretical model suitable for explaining the self-reported hand hygiene behavior of hospital patients and visitors. This was achieved by conducting a survey in four German hospitals using questionnaires based on the three theoretical models: TPB, HAPA, and TDF. All three models proved useful for examining self-reported hand hygiene practice in hospitals. Among patients, 52% of the variance in hand hygiene behavior during their hospital stay was accounted for by the TDF domains, 44% by the modified HAPA model, and 40% by the TPB. Among visitors, these figures were 59% (TDF), 37% (HAPA), and 55% (TPB) of explained variance in hand hygiene before and after patient contact.

#### HAPA

The original HAPA path model did not fit the patient and visitor data well. According to the HAPA model, action and coping planning act as mediators between intention and behavior [32, 58]. However, these planning processes did not emerge as mediators in the present study. Our first assumption on why the planning processes did not fit in the model had to do with the lack of focus on patients’ and visitors’ hand hygiene behavior for infection prevention. Researchers and hospital hygiene specialists have only recently begun to pay more attention to patients and visitors as a potential vector for transmitting pathogens. Therefore, attempts to include them in the hospital’s infection prevention strategy are still at an early stage. Consequently, we expected that many participants would be in the pre-intentional phase of the HAPA model because they might lack awareness that they should clean their hands. The HAPA questionnaire included a state of change item also used in previous research [54]. Surprisingly, most patients (73.4%) and visitors (80.3%) positioned themselves in the post-intentional action phase. This finding corresponds to their high level of self-reported hand hygiene behavior and indicates that patients and visitors are aware that they should clean their hands regularly in hospitals. However, this finding makes it harder to explain why the planning constructs seemed irrelevant for this target group. A second explanation could lie in the cross-sectional nature of the study. Some of the planning items convey more meaning in longitudinal research, where participants try to change their behavior deliberately. Therefore, the HAPA model should be reexamined within a longitudinal behavior change intervention. A third explanation might be the nature of the behavior, as already mentioned in the results section. Being hospitalized is usually a straining and anxiety-inducing situation for patients and their relatives, during which hand hygiene might not be a priority for them. Consequently, patients and visitors probably do not plan for “when”, “where”, and “how” to clean their hands, nor for how to overcome barriers. It is intuitively plausible that the planning constructs do not fit in the model for this behavior and target group. Including action and coping planning as determinants for behavior might only be relevant if people are motivated to change. After the modifications to the model, it fits both the patient and visitor data well, but it still explained less variance in the self-reported hand hygiene behavior than the TDF model.

#### TPB and TDF

The path structure of the TPB and the TDF did not need any changes. TDF was created “to simplify and integrate a plethora of behavior change theories and make theory more accessible to, and usable by, other disciplines” [27: p.2]. It is no causal model of behavior and does not include mediation pathways, which would indicate a causal direction of how its domains are related to each other and the behavior in question. The model fit of a just-identified model with equal numbers of variables and parameters with a unique solution is inevitably perfect, and the results are identical with a linear multiple regression analysis. To compare the fit of the TPB and TDF, we fixed a non-significant parameter to zero. Both the proposed TPB and the TDF model fitted the patient and visitor data very well. When comparing all fit indices, the TDF showed a slightly better fit. Additionally, the TDF explained more variance in self-reported hand hygiene behavior than the TPB in both samples. Thus, it can be concluded that both models are suitable for explaining hand hygiene behavior among hospital patients and visitors. Still, the more comprehensive TDF would be our model of choice to determine barriers and levers related to patients’ and visitors’ hand hygiene in healthcare facilities, and to use as a base for designing interventions.

### Summary aim two: Detecting critical determinants

The second aim of the study was to find critical determinants of patients’ and visitors’ hand hygiene behavior. This was achieved by analyzing the correlations between the proposed factors and identifying the most relevant predictors for self-reported behavior.

#### TPB

In both samples, all the pre-intentional TPB-variables significantly correlated with people’s intention to sanitize their hands. The data showed that especially PBC played an essential role. The associations between intention and behavior as well as PBC and behavior were significant in both samples. However, among patients, the indirect effect between PBC and behavior was stronger than the direct effect, while the opposite was true for visitors. This might imply that intention formation is more important for patients than visitors. For visitors, the ease or difficulty of hand hygiene (e.g., access to dispensers) was the most relevant direct predictor for the behavior. But for patients, who have more indications to sanitize their hands throughout the day, the ease or difficulty of cleaning one's hands might lead to the formation of an explicit intention for whether it is worth bothering to engage in the behavior.

#### HAPA

Patients’ intention to sanitize their hands significantly correlated with the variables of self-efficacy, positive outcome expectations, perceived severity of harm, environmental resources, and action control. Self-efficacy and action control had the most substantial effects. Patients’ hand hygiene behavior correlated positively with intention and action control. Overall, these results are in line with findings from a previous study that showed self-efficacy and outcome expectancies to be connected with handwashing intention [33]. Likewise, the study reported intention and especially action control to be associated with hand hygiene behavior [33]. Among visitors, self-efficacy, positive outcome expectations, and action control also significantly correlated with the intention. Again, self-efficacy and especially action control had the most substantial effects. Other than the patients, the perceived likelihood but not the perceived severity was associated with intention. The only significant correlates for hand hygiene behavior among visitors was intention. The association between action control and behavior was mediated by intention. While intention was the strongest predictor for both samples' behavior, the intention-behavior gap was not fully bridged by self-efficacy and action control (and planning processes, which did not fit in the model).

#### TDF

Patients’ hand hygiene behavior significantly correlated with the domains of social/professional role and identity; motivation and goals; memory, attention, and decision processes; and emotions. For visitors, the significant predictors were social/professional role and identity; memory, attention, and decision processes; and knowledge and skills; as well as emotions. The only difference was that instead of motivation and goals, knowledge and skills were associated with visitors’ behavior. In both samples, the social/professional role and identity domain had the largest effect on behavior. The memory, attention, and decision processes domain also had a substantial effect on hand hygiene behavior among both patients and visitors. When comparing our results with findings from a Canadian qualitative study that looked at patients’ hand hygiene behavior [17], we can see that the memory, attention, and decision processes domain emerged as a critical factor in both studies. The other study identified the social influences domain as an essential factor, while we found the social/professional role and identity domain to be a relevant predictor. The two domains are connected, since both have social norms as an underlying process. More theoretical clarity about the distinction between the two domains might be needed. Finally, in our sample, the environmental context and resources domain was not relevant in either group, but has been identified as an important barrier to patients’ hand hygiene behavior in the other study [17]. While a lack of products or not recognizing hand rub as such was identified as a problem in the Canadian study, most participants in our sample did not indicate that a lack of resources was an issue. It remains unclear if the healthcare systems or hospitals in which the respective data was collected are responsible for this mismatch.

In conclusion, both the data from the TPB and the HAPA model showed that behavioral intention is a strong but not perfect predictor for self-reported hand hygiene behavior. These results underline the importance of intention formation to explain behavior and facilitate behavior change. However, the findings also show that we need a better understanding of the psychological processes determining intention and leading from intention to action. When looking only at the strongest and most coherent determinants affecting self-reported hand hygiene behavior directly or indirectly via intention, we found that they can be assigned to two broad clusters. The first cluster includes the model constructs PBC; self-efficacy; action control; and memory, attention, and decision processes. Self-regulatory processes are at the core of all three constructs. The second cluster includes the constructs of subjective norm and role and identity. Social influence processes, especially norms, are at the heart of both constructs.

### Summary goal three: Comparing patients and visitors with healthcare professionals

The third and final aim of the present study was to examine whether critical determinants for hand hygiene behavior in hospitals differ between healthcare professionals and non-professionals. This was achieved by drawing a qualitative comparison between our survey results and previous research on hand hygiene among healthcare workers. It should be noted that this was not a standardized, quantitative comparison of effect sizes. Some studies used a qualitative method; therefore, comparing effect sizes was not possible.

#### TPB

The TPB is the most widely used theory to identify determinants for hand hygiene behavior in the literature. Some but not all of the studies of healthcare worker reported all three pre-intentional variables (i.e., attitude, subjective norm, and PBC) as relevant predictors for self-reported hand hygiene compliance [38–40], which is in line with our results. In general, PBC consistently emerged as a critical determinant for healthcare workers’ hand hygiene behavior [30, 38–44]. Again, this corresponds well with the present study’s results, where PBC also emerged as the most influential factor for patients’ and visitors’ hand hygiene behavior within the TPB.

#### HAPA

To our knowledge, the only other research project that applied the HAPA to investigate hand hygiene behavior in hospitals was the PSYGIENE project [46, 47]. Unfortunately, the study, which looked at the pre-intentional HAPA variables, did not report correlations with self-reported hand hygiene compliance [46]. The second study, which included the post-intentional HAPA variables, found that compliance among physicians was associated with environmental resources, maintenance self-efficacy, and action control. Self-reported compliance among nurses was only linked to action control. Combined with the PSYGIENE results, we can see action control to be the factor most strongly associated with self-reported hand hygiene behavior or intention across healthcare professionals and non-professionals. This result is in line with other research [33] that also identified action control as the primary determinant for hand hygiene behavior outside the healthcare context.

#### TDF

Three studies identified facilitators and barriers of healthcare workers’ hand hygiene behavior, according to TDF [35, 48, 49]. Like these studies, we also found memory, attention, and decision processes to be among the most crucial barriers to adequate hand hygiene in hospitals among visitors and patients. Contrary to the studies of healthcare workers, we did not find the knowledge and skills domain to be critical for patients’ and visitors’ hand hygiene behavior. In one of the healthcare worker-studies [35], the method itself of asking people about their hand hygiene behavior only when they made a mistake seemed to unveil to them that they did not know the appropriate behavior according to the guidelines for the situation. In the other two studies, knowledge and skills was among the less influential determinants [48, 49]. Future research should objectively measure the domain knowledge amongst laypeople to test how important the domain really is. Another consistent barrier to healthcare workers’ hand hygiene compliance was the environmental context and resources domain (mainly lack of time and accessibility of products). We did not identify this domain as a relevant barrier for patients’ and visitors’ hand hygiene. This is plausible since patients and visitors are most likely not constrained by time pressure. Additionally, the availability of hand hygiene products was good in all hospitals in our study. In line with our results, the social/professional role and identity domain was identified as an essential determinant for hand hygiene in two healthcare worker-studies [48, 49]. In the other study [35], participants’ verbal explanations for non-compliance with guidelines were recorded and coded. It is plausible that healthcare workers do not link individual cases of non-compliance with their general professional identity at the moment of the event. This last point highlights that the method by which facilitators and barriers of hand hygiene behavior are measured might have an influence on which determinants will surface as being important. Therefore, the responses patients and visitors give for cleaning or not cleaning their hands in an open-answer format should be compared to the results from the questionnaire findings.

Overall, the critical determinants for healthcare workers’ hand hygiene behavior published in the literature are similar to the ones we found for hospital patients and visitors. Some differences between the two target groups might be explained by the method used to measure the model’s components and by the studies’ designs. However, considering the similarities, we think that successful intervention strategies which improve healthcare workers’ hand hygiene behavior might also be useful for targeting patients and visitors.

### Limitations

The present study had some limitations. First, all data, including the data that constituted the dependent variable, were self-reported. Previous research has shown that self-reported hand hygiene often only correlates weakly with actually observable behavior and is usually overrated [e.g., 30, 59]. To our knowledge, within the hospital environment, the gap between self-reported and observable behavior has been demonstrated only for healthcare workers. It is unclear if patients and visitors are as prone to overreport their hand hygiene behavior in hospitals. But handwashing rates after using the restroom outside hospitals showed that actual rates were significantly lower than self-reported rates [e.g., 60]. Nevertheless, using self-reported behavior is a pragmatic and economical way to gather data for large samples in hospitals. Many previous studies have also used self-reported hand hygiene behavior to investigate potential facilitators and barriers (see for instance Table 4). Further research should verify our results using observed behavior as the outcome measure. Second, we employed a cross-sectional study design. This means that no statements about causal effects can be made. One drawback of path diagrams is that they imply a causal direction, which cannot be verified with a cross-sectional dataset. All associations between hand hygiene behavior and model variables are bivariate correlations. But there is longitudinal research on both the TPB and HAPA, indicating the reliability of the described path directions [58, 61–63]. Third, while the internal consistency of most scales ranged between excellent and acceptable, some scales did not meet a Cronbach’s alpha of at least .70. However, Cronbach’s alpha depends on the number of items included in the scale and is often low when the scale consists of only a few items. In this case, using the mean inter-item correlation is more appropriate. In all cases where Cronbach’s alpha was below .70, the mean inter-item correlation was within the recommended boundaries of .20 - .50. The CFAs showed that the items for two scales (HAPA_Visitors_: risk perception likelihood and TDF_Visitors_: environmental context) did not load well on one factor, and single items representing the concept the best were included in the model. Measuring perceived risk likelihood with a single item is consistent with some other published research [46, 64]. However, the more complex behavioral domain of environmental context is probably not captured in its entirety with only one item. The bivariate correlation for environmental context did not differ strongly between patients and visitors. Therefore, the results would probably not have fundamentally changed, even when the scale had better psychometric qualities. Nevertheless, several of the HAPA variables and TDF domains were only measured with two items, which is probably not ideal and will only capture a narrow bandwidth of the construct. Further research should be conducted to improve the quality of some of these scales for non-healthcare professionals. Finally, even though we collected data in four different hospitals ranging from a small countryside clinic to a large university hospital, all of them were in Germany and within a 100km radius of each other. Therefore, we cannot tell if the conclusions drawn from our data could be generalized for other countries.

### Practical implications

The present study results have several implications for designing behavior change interventions to improve hospital patients’ and visitors’ hand hygiene behavior. The results indicate that model constructs related to self-regulatory processes are important determinants for hand hygiene behavior. To reduce the need for self-regulation (i.e., self-monitoring and managing behavior), hospitals could change the environment to nudge people to clean their hands regularly. For instance, placing dispensers at highly visible and accessible locations makes it easier for people to use the dispenser and, therefore, might improve PBC and self-efficacy. This approach has been shown to increase dispenser usage rates among patients and visitors as well as healthcare workers [15, 65–67]. Another method to raise awareness about hand hygiene and prevent forgetting is to install prominent signs and reminders close to hand-rub dispensers [66, 68–71]. Technically supported interventions employing attention-grabbing visual and auditory reminders to motivate people to clean their hands have shown promising results for improving hand hygiene behavior in hospitals [72, 73]. However, auditory reminders should be used with caution because they might increase noise pollution and alert fatigue. Yet another option to reduce the need for self-regulation from patients and visitors to clean their hands would be to implement a form of compulsory hand hygiene, such as having nurses apply hand rub to patients before meals. This approach has been shown to be an effective way to reduce healthcare-associated infections in a couple of studies [10, 11, 74]. However, compulsory hand hygiene programs require additional human resources and might therefore not be feasible for most facilities. Finally, interventions could be designed to improve patients’ and visitors’ self-regulatory processes by boosting their self-efficacy and PBC. A previous study has shown that participants who created specific hand-washing action plans and were reminded about their ability to comply with these plans and about their past successes increased their handwashing frequency [61].

The present study’s results also indicate that targeting social influence processes, especially norms, might be a promising approach for a behavior change intervention to improve hand hygiene behavior in hospitals. Patients and visitors reported cleaning their hands more often if they felt it was their responsibility to play an active role in preventing infections and that other people expected them to do it. Therefore, interventions should be designed to convey this idea. Informational materials, signs, and other reminders should include normative messages to highlight the importance of patients’ and visitors' roles in infection prevention. Previous studies have shown that employing intervention material that utilizes social influence processes can increase the hand hygiene rate in healthcare facilities [71, 72].

## Conclusion

In conclusion, the present study is the first to systematically compare three theoretical models, TPB, HAPA, and TDF, on their usefulness for explaining hospital patients’ and visitors’ hand hygiene practices. The TDF accounted for the largest share of variance in the self-reported behavior and showed excellent model fit. Two clusters of variables emerged as important determinants for hospital patients’ and visitors’ hand hygiene behavior: self-regulatory processes and social influence processes. Overall, the determinants for hand hygiene behavior are similar for healthcare professionals (according to the literature) and non-professionals. Therefore, patients and visitors can be included in the infection prevention strategy without substantial changes to the action plans. The results of the present study should help hospital hygiene practitioners to design and evaluate future interventions to improve patients’ and visitors’ hand hygiene behavior in healthcare facilities. Better hand hygiene practice should help to reduce the rates of healthcare-associated infections and improve patient safety.

## Supporting information

S1 Table Overview of key measures and psychometric data of the three questionnaires for both patients and visitors. *Note*. # = number of items included in the scale, α = Cronbach’s alpha, IIC = mean inter-item correlation, *M* = mean, *SD* = standard deviation.
(PDF)

S2 Table Coefficients for the TDF path models with the parameter for environmental context and resources fixed to zero. *Note*. β = standardized coefficient, *SE* = standard error; *z* = β */SE*, *p* = probability value, LL = lower limit, UP = upper limit.
(PDF)
