# Supplementary material for: Hand(y) hygiene insights: Applying three theoretical models to investigate hospital patients’ and visitors’ hand hygiene behavior
Source: PLoS One. 2021 Jan 14;16(1):e0245543. doi: 10.1371/journal.pone.0245543 (PMC7808666; doi:10.1371/journal.pone.0245543)
Supplement: S1 Table — Note. # = number of items included in the scale, α = Cronbach’s alpha, IIC = mean inter-item correlation, M = mean, SD = standard deviation. (PDF) [file pone.0245543.s001.pdf]

S1 Table. Overview of key measures and psychometric data of the three questionnaires for both patients and visitors.

Theory	Construct	Target Group	Item Example	Scale	# of Items	α/IIC	*M*	*SD*
TPB	Attitude	Patients	"As a hospital patient, regular sanitation is…"	1 = useless; 7 = useful	9	.83/.40	6.33	0.79
		Visitors	"As a hospital visitor, hand sanitation is…"		5	.84/.59	6.55	0.91
	Subjective Norms	Patients	"As a patient, I am expected to regularly sanitize my hands in the hospital."	1 = strongly disagree; 7 = strongly agree	11	.90/.44	4.96	1.40
		Visitors	"As a visitor, I am expected to sanitize my hands in the hospital."		4	.79/.46	5.40	1.47
	Perceived Behavioral Control (PBC)	Patients	"I am confident that I manage to clean my hands in the hospital regularly if I want to."	1 = strongly disagree; 7 = strongly agree	10	.77/.27	5.66	0.99
		Visitors	"It is easy for me to sanitize my hands in the hospital."		5	.74/.38	5.48	1.34
	Intention	Patients	"I intend to clean my hands in the hospital before eating."	1 = strongly disagree; 7 = strongly agree	7	.82/.40	5.68	1.20
		Visitors	"I intend to sanitize my hands before touching a patient."		2	.81/.57	6.16	1.27
	Behavior	Patients	"I sanitize my hands before I eat in the hospital."	1 = never; 6 = always	8	.88/.46	4.07	1.23
		Visitors	"I sanitize my hands before contact with a patient."	1 = rarely; 5 = always	2	.83/.78	3.82	1.20

Theory	Construct	Target Group	Item Example	Scale	# of Items	α/IIC	*M*	*SD*
HAPA	Risk Perception (Likelihood)	Patients	"In your opinion, how likely is it that another patient here in the hospital becomes infected with a pathogen?"	1 = very unlikely 7 = very likely	4	.78/.40	4.28	1.48
		Visitors	"In your opinion, how likely are you to transmit pathogens within the hospital if you do not sanitize your hands?"		1	--	4.78	1.73
	Risk Perception (Severity)	Patients	"How severe is an infection with a pathogen for the health of another patient?"	1 = not severe 7 = very severe	3	.89/.66	5.50	1.36
		Visitors	"How severe is an infection with a pathogen for the health of a patient?"		2	.66/.42	5.49	1.32
	Outcome Expectancies (Positive)	Patients	"If I regularly clean my hands in the hospital, I help to prevent infections."	1 = strongly disagree; 7 = strongly agree	3	.85/.67	6.48	1.02
		Visitors	"If I sanitize my hands in the hospital, I help to prevent infections."		2	.65/.47	6.12	1.28
	Outcome Expectancies (Negative)	Patients	"If I regularly clean my hands in the hospital, I get skin problems on my hands."	1 = strongly disagree; 7 = strongly agree	4	.74/.41	2.05	1.29
		Visitors	"If I sanitize my hands in the hospital, I get skin problems on my hands."		4	.68/.41	1.88	1.11
	Self-Efficacy	Patients	"I am confident that I can engage in hand hygiene in the hospital, even if people present do not do this."	1 = strongly disagree; 7 = strongly agree	5	.87/.56	5.86	1.36
		Visitors	"I am confident that I can clean my hands in the hospital, even if people present do not do this."		6	.89/.54	5.89	1.31

Theory	Construct	Target Group	Item Example	Scale	# of Items	α/IIC	*M*	*SD*
HAPA	Intention	Patients	"I intend to clean my hands in the hospital before eating."	1 = strongly disagree; 7 = strongly agree	7	.84/.38	5.72	1.18
		Visitors	"I intend to sanitize my hands before touching a patient."		2	.81/.61	5.74	1.58
	Planning	Patients	"I have been planning how to deal with obstacles and events that make it difficult for me to clean my hands in the hospital."	1 = strongly disagree; 7 = strongly agree	2	.74/.53	3.96	1.86
		Visitors	"I have been planning how to deal with obstacles and events that make it difficult for me to clean my hands in the hospital."		2	.70/.47	3.73	1.87
	Resources	Patients	"The hospital is equipped with a sufficient number of hand-rub dispensers."	1 = strongly disagree; 7 = strongly agree	3	.82/.56	5.68	1.33
		Visitors	"The hospital is equipped with a sufficient number of hand-rub dispensers."		2	.75/.51	5.17	1.56
	Action Control	Patients	"I make sure that I regularly engage in hand hygiene in the hospital."	1 = strongly disagree; 7 = strongly agree	3	.76/.49	5.57	1.38
		Visitors	"I know exactly when to sanitize my hands in the hospital as a visitor."		2	.65/.46	5.87	1.36
	Behavior	Patients	"Before I eat in the hospital, I sanitize my hands."	1 = never; 6 = always	8	.89/.44	4.14	1.24
		Visitors	"I sanitize my hands before contact with a patient."	1 = rarely; 5 = always	2	.83/.65	3.59	1.32

Theory	Construct	Target Group	Item Example	Scale	# of Items	α/IIC	*M*	*SD*
TDF	Knowledge and Skills	Patients	“Hand hygiene training is available to me as a patient.”	1 = strongly disagree; 7 = strongly agree	5	.76/.37	5.87	1.15
		Visitors	“As a visitor, I can have explained to me how correct hand hygiene behavior works in the hospital.		4	.63/.27	4.97	1.34
	Role and Identity	Patients	“As a patient, I engage in hand hygiene in the hospital out of respect for the other patients.”	1 = strongly disagree; 7 = strongly agree	3	.85/.56	5.85	1.36
		Visitors	“As a visitor, I engage in hand hygiene in the hospital out of respect for the patients.”		3	.76/.47	5.74	1.45
	Beliefs about Capabilities	Patients	“I am confident in my ability to clean my hands regularly if I want to.”	1 = strongly disagree; 7 = strongly agree	2	.52/.31	2.97	1.80
		Visitors	“I am uncertain when to sanitize my hands in the hospital as a visitor.”		3	.66/.34	2.32	1.42
	Beliefs about Consequences	Patients	“If I do not engage in regular hand hygiene in the hospital, I risk catching an infection myself.”	1 = strongly disagree; 7 = strongly agree	4	.80/.51	5.31	1.46
		Visitors	“If I do not sanitize my hands in the hospital, I risk catching an infection myself.”		2	.60/.36	5.74	1.51
	Motivation and Goals	Patients	“I cannot be bothered with regular hand hygiene in the hospital.” (inverted)	1 = strongly disagree; 7 = strongly agree	6	.75/.38	5.91	1.05
		Visitors	“I cannot be bothered to sanitize my hands in the hospital.” (inverted)		2	.42/.32	1.93	1.27

Theory	Construct	Target Group	Item Example	Scale	# of Items	α/IIC	*M*	*SD*
TDF	Memory, Attention, and Decision Processes	Patients	"Sometimes I leave out hand hygiene in the hospital simply because I forget it."	1 = strongly disagree; 7 = strongly agree	5	.87/.49	3.93	1.78
		Visitors	"Sometimes I forget to disinfect my hands in the hospital."		4	.66/.28	3.39	1.60
	Environmental Context (Barriers)	Patients	"Hand-rub dispensers are easy to miss in the hospital."	1 = strongly disagree; 7 = strongly agree	3	.65/.39	2.36	1.35
		Visitors	"Hand-rub dispensers are hard to find in the hospital."		1	--	3.25	2.04
	Social Influences	Patients	"I engage in hand hygiene in the hospital because I do not want to disappoint the expectations of others."	1 = strongly disagree; 7 = strongly agree	4	.60/.22	2.63	1.26
		Visitors	"I am encouraged by other people (e.g. other visitors, patients, or hospital staff) to sanitize my hands."		3	.68/.35	2.41	1.43
	Emotion	Patients	"I feel angry if hand hygiene is not carried out by others (e.g., other patients, visitors, or staff members)."	1 = strongly disagree; 7 = strongly agree	3	.71/.38	4.43	1.78
		Visitors	"I feel angry if hand hygiene is not carried out by others (e.g., other visitors, patients, or staff members)."		4	.72/.32	4.14	1.69

**Theory**	**Construct**	**Target Group**	**Item Example**	**Scale**	**# of Items**	**α/IIC**	***M***	***SD***
TDF	Behavioral Regulations (Action Plans)	Patients	"Hospital interventions to improve hand hygiene have influenced me to clean my hands in the hospital more often."	1 = strongly disagree; 7 = strongly agree	3	.80/.50	4.49	1.92
		Visitors	"Hospital interventions to improve hand hygiene have influenced me to clean my hands in the hospital more often."		3	.71/.37	3.95	1.82
	Behavior	Patients	"Before I eat in the hospital, I sanitize my hands."	1 = never; 6 = always	8	.90/.46	4.04	1.25
		Visitors	"I sanitize my hands before contact with a patient."	1 = rarely; 5 = always	2	.83/.66	3.72	1.31

Note. # = number of items included in the scale, α = Cronbach's alpha, IIC = mean inter-item correlation, *M* = mean, *SD* = standard deviation
