# Supplementary material for: Hand(y) hygiene insights: Applying three theoretical models to investigate hospital patients’ and visitors’ hand hygiene behavior
Source: PLoS One. 2021 Jan 14;16(1):e0245543. doi: 10.1371/journal.pone.0245543 (PMC7808666; doi:10.1371/journal.pone.0245543)
Supplement: S2 Table — Note. β = standardized coefficient, SE = standard error; z = β /SE, p = probability value, LL = lower limit, UP = upper limit. (PDF) [file pone.0245543.s002.pdf]

S2 Table. Coefficients for the TDF path models with the parameter for environmental context and resources fixed to zero

	Path	β	*SE*	*z*	*p*	95% CI for β LL	95% CI for β UL
Patients	Knowledge/Skills → Behavior	0.02	0.05	0.40	.686	-0.08	0.12
	Role and Identity → Behavior	0.30	0.04	6.85	<.001	0.21	0.39
	Capability → Behavior	0.03	0.05	0.61	.540	-0.07	0.13
	Consequences → Behavior	0.04	0.05	0.82	.415	-0.06	0.15
	Motivation/Goals → Behavior	0.16	0.06	2.82	.005	0.05	0.28
	Memory/Attention → Behavior	-0.19	0.05	-3.61	<.001	-0.30	-0.09
	Environment → Behavior	0.00	0.00	NA	NA	0.00	0.00
	Social Influences → Behavior	0.08	0.05	1.62	.106	-0.02	0.18
	Emotions → Behavior	0.23	0.06	3.96	<.001	0.12	0.34
	Beh. Regulations → Behavior	0.04	0.06	0.73	.468	-0.08	0.16
Visitors	Knowledge/Skills → Behavior	0.13	0.05	2.59	.010	0.03	0.22
	Role and Identity → Behavior	0.39	0.06	6.22	<.001	0.26	0.51
	Capability → Behavior	0.09	0.05	1.60	.109	-0.02	0.19
	Consequences → Behavior	0.07	0.05	1.39	.165	-0.03	0.18
	Motivation/Goals → Behavior	0.04	0.05	0.73	.463	-0.06	0.14
	Memory/Attention → Behavior	-0.39	0.05	-7.67	<.001	-0.49	-0.29
	Environment → Behavior	0.00	0.00	NA	NA	0.00	0.00
	Social Influences → Behavior	0.08	0.05	1.59	.111	-0.02	0.17
	Emotions → Behavior	0.13	0.06	2.39	.017	0.02	0.24
	Beh. Regulations → Behavior	-0.08	0.05	-1.64	.101	-0.18	0.02

Note. β = standardized coefficient, *SE* = standard error; *z* = β /*SE*, *p* = probability value, LL = lower limit, UP = upper limit.
